# The Role of the NADPH Oxidase NOX2 in Prion Pathogenesis

**DOI:** 10.1371/journal.ppat.1004531

**Published:** 2014-12-11

**Authors:** Silvia Sorce, Mario Nuvolone, Annika Keller, Jeppe Falsig, Ahmet Varol, Petra Schwarz, Monika Bieri, Herbert Budka, Adriano Aguzzi

**Affiliations:** 1 Institute of Neuropathology, University Hospital of Zurich, Zurich, Switzerland; 2 Institute of Surgical Pathology, University Hospital of Zurich, Zurich, Switzerland; University of Florida, United States of America

## Abstract

Prion infections cause neurodegeneration, which often goes along with oxidative stress. However, the cellular source of reactive oxygen species (ROS) and their pathogenetic significance are unclear. Here we analyzed the contribution of NOX2, a prominent NADPH oxidase, to prion diseases. We found that NOX2 is markedly upregulated in microglia within affected brain regions of patients with Creutzfeldt-Jakob disease (CJD). Similarly, NOX2 expression was upregulated in prion-inoculated mouse brains and in murine cerebellar organotypic cultured slices (COCS). We then removed microglia from COCS using a ganciclovir-dependent lineage ablation strategy. NOX2 became undetectable in ganciclovir-treated COCS, confirming its microglial origin. Upon challenge with prions, NOX2-deficient mice showed delayed onset of motor deficits and a modest, but significant prolongation of survival. Dihydroethidium assays demonstrated a conspicuous ROS burst at the terminal stage of disease in wild-type mice, but not in NOX2-ablated mice. Interestingly, the improved motor performance in NOX2 deficient mice was already measurable at earlier stages of the disease, between 13 and 16 weeks post-inoculation. We conclude that NOX2 is a major source of ROS in prion diseases and can affect prion pathogenesis.

## Author Summary

The deposition of misfolded, aggregated prion protein in the brain causes transmissible spongiform encephalopathies (TSE), a group of disorders including Creutzfeldt–Jakob disease and mad cow disease. TSE are characterized by neurodegeneration and progressive, lethal neurological dysfunction. Signs of oxidative damage are found in TSE, implying excessive production of reactive oxygen species (ROS), yet their source is unclear. Here, we analyzed the role of the NADPH oxidase enzyme, NOX2, in prion pathogenesis. NOX2 is a membrane-bound electrochemical pump that generates ROS. We found that NOX2 is upregulated in the brains of patients with Creutzfeldt-Jakob disease and of prion-infected mice. Interestingly, NOX2 ablation led to abrogation of ROS production in mice inoculated with prions, and was associated with a milder clinical course of the disease and increased life expectancy. We conclude that NOX2 is a relevant contributor to the excessive production of ROS. This study spawns the possibility that inhibiting NOX2 activation might help attenuate prion disease progression – a legitimate and important goal even if there is little reason to expect anti-NOX2 therapies to be curative.

## Introduction

Prion infections cause the deposition of misfolded, aggregated prion protein (PrP^Sc^) in the brain, and lead to progressive, lethal neurodegeneration. These diseases are also known as transmissible spongiform encephalopathies because their main neuropathological hallmark is the presence of vacuoles in affected brain regions [1]. Some of the molecular mechanisms underlying these processes are beginning to be understood, but it is still impossible to arrest the progression of the disease. Expression of prion protein on neuronal cell membranes is a prerequisite to neurodegeneration [2], [3], and only the genetic ablation of prion protein has conferred complete resistance to the disease [4]. In contrast, pharmacological and genetic approaches targeting mediators of prion-dependent neurotoxicity pathways have been found to modify the course of the disease, but never to reverse it [5]–[7]. We posit that the identification of additional molecular components involved in prion neurotoxicity will be instrumental in designing effective therapeutic approaches.

Signs of oxidative damage have been described in patients affected by CJD and in experimental models of prion disease [8]–[11]. Also, an accelerated course of the disease has been recently reported in mice devoid of proteins involved in oxidant defense mechanisms (SOD1, OGG1 and MUTYH), suggesting a deleterious effect of increased oxidative stress levels [12], [13]. However, the source of excessive ROS production has not been yet described, and the impact of reduced oxidative stress levels on the course of the disease remains unclear.

The NADPH oxidase enzyme NOX2 is an electron transporter whose only known physiological function is the production of ROS [14]. NOX2 produces ROS by the interaction with the transmembrane protein, p22^phox^, the cytosolic subunits, p47^phox^, p67^phox^, p40^phox^, and one of the small Rho GTP-binding proteins, Rac1 or Rac2. This enzymatic complex plays an important role in phagocytes, where production of ROS is necessary for effective host defense [14]. However, in certain pathological situations, an excessive activation of NOX2 can lead to oxidative imbalance and contribute to disease progression [14], [15].

We have recently reported that NOX2 is involved in the neurotoxicity of ligands targeting the prion protein, PrP^C^ [16]. Antibodies to the globular domain of PrP^C^, but not to other epitopes, elicited rapid neurotoxicity *in vivo* and *in vitro*. After administration of such neurotoxic antibodies, NOX2-deficient mice developed smaller lesions than wild-type controls, suggesting a role of NOX2 in PrP^C^-dependent neurotoxicity processes.

Such mechanisms could also be involved in the pathobiology of prion diseases. We therefore investigated the role of NOX2 in *bona fide* prion disorders. We found that NOX2 is mainly expressed in microglia, particularly in the cells surrounding spongiform vacuoles in brains of CJD patients. NOX2-deficiency in mice resulted in decreased ROS production and reduced spongiform changes at the terminal phase of the disease. These effects were associated with an increase in survival and, more importantly, with improved motor function during the clinical phase of the disease. Hence NOX2 is a major contributor to the excessive production of ROS detected in prion disorders, and therefore influences prion pathogenesis.

## Results

### Expression of NOX2 in CJD patients

We first investigated the expression and localization of NOX2 in brain samples of patients affected by CJD. Immunohistochemistry was performed on sections of cerebellum and frontal cortex from ten CJD patients with a balanced distribution of PrP types 1 and 2 as reported on Western blots of PK-treated brain homogenates (S1 Table). Samples of three patients affected by Alzheimer's disease (AD) were used as controls (CERAD definite and Braak & Braak stage V); the first patient also presented with the cortical type of diffuse Lewy body disease.

**Figure 1:**
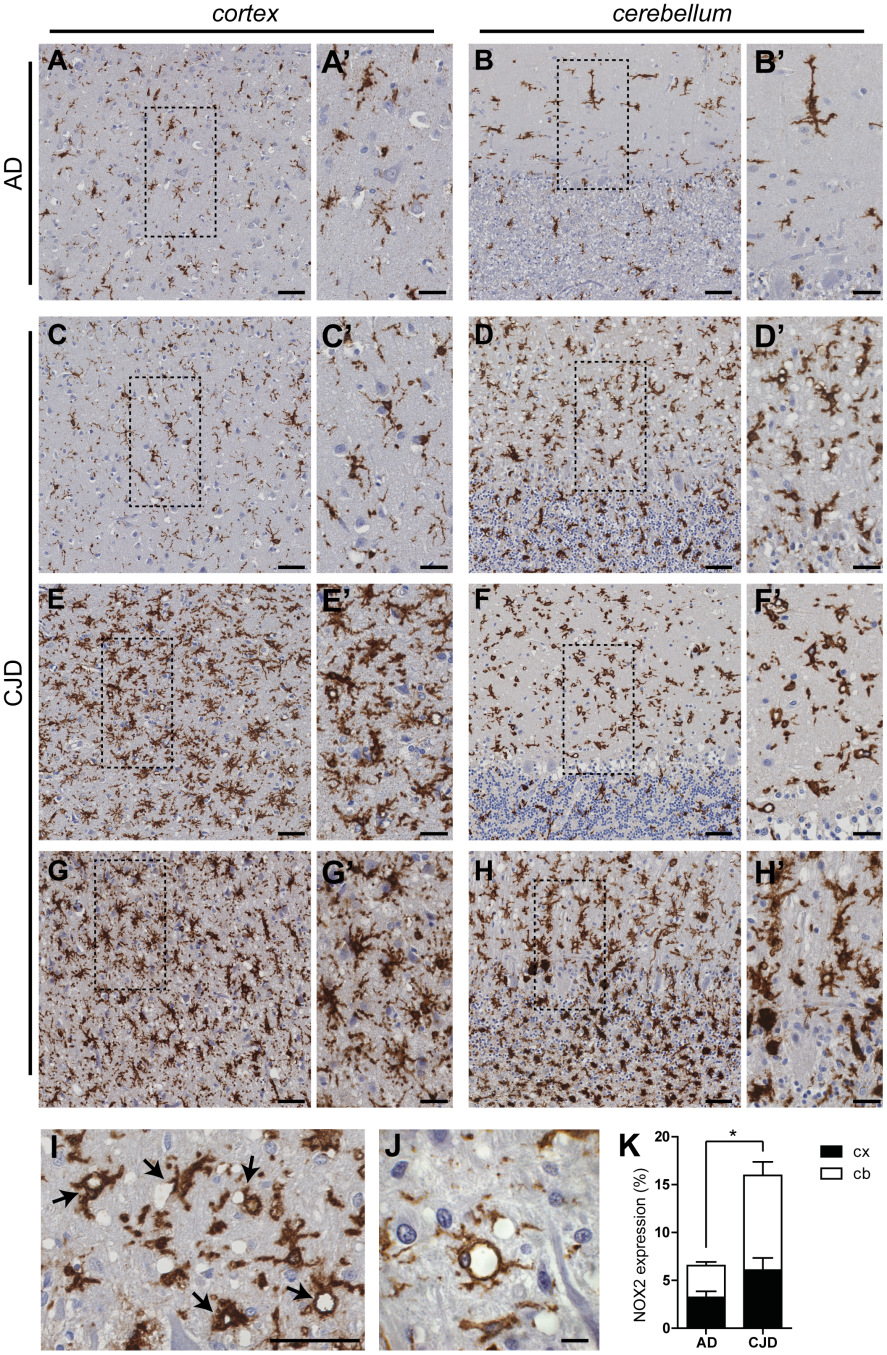
NOX2 expression is increased in affected brain regions of CJD patients. The expression of NOX2 was analyzed by immunohistochemistry in paraffin-embedded sections of cortical (**A**,**C**,**E**,**G**) and cerebellar (**B**,**D**,**F**,**H**) areas of AD (A–B) and CJD (C–H) patients. Representative images of CJD brains with predominant cerebellar (C–D) or cortical (E–F) NOX2 expression are shown together with images of CJD brains with increased NOX2 staining in both areas (G–H). Areas highlighted with dashed boxes in pictures A to H are reproduced at higher magnification in images A′ to H′. Intense NOX2 staining was present around the rim of spongiform vacuoles (**I–J**; indicated by black arrows in I). Scale bar: 50 µm (A–H); 25 µm (A′–H′); 50 µm (I); 10 µm (J). (**K**) Bar graphs representing mean ± SEM NOX2 protein expression, quantified as the percentage of the surface occupied by the NOX2 staining over the total measured area in selected regions of frontal cortex (cx) and cerebellar cortex (cb) of AD and CJD patients (AD, n = 3; CJD, n = 10; *P = 0.0398; Student's t test).

The specificity of the anti-human NOX2 antibody was confirmed by the detection of the appropriate band in Western blotting of PLB-985 human myeloid cells and concomitant absence of signal in human NOX2-deficient PLB-985 cells [17], human NOX1-overexpressing CHO cells and HEK293 cells overexpressing human NOX3, 4, 5, DUOX1 and DUOX2 (personal communication, Dr. Vincent Jaquet, University of Geneva). As indicated in representative images (Fig. 1A–H), conspicuous NOX2 staining was detected in affected brain regions of CJD patients. Increased expression of NOX2 in CJD, as compared to AD brain sections, was confirmed by quantitative analysis (Fig. 1K). The morphology of NOX2-positive cells suggested that they consisted mainly, if not exclusively, of microglia. In all CJD brains we noticed a particular strong expression of NOX2 around a subset of vacuoles (Fig. 1I–J).

**Figure 2:**
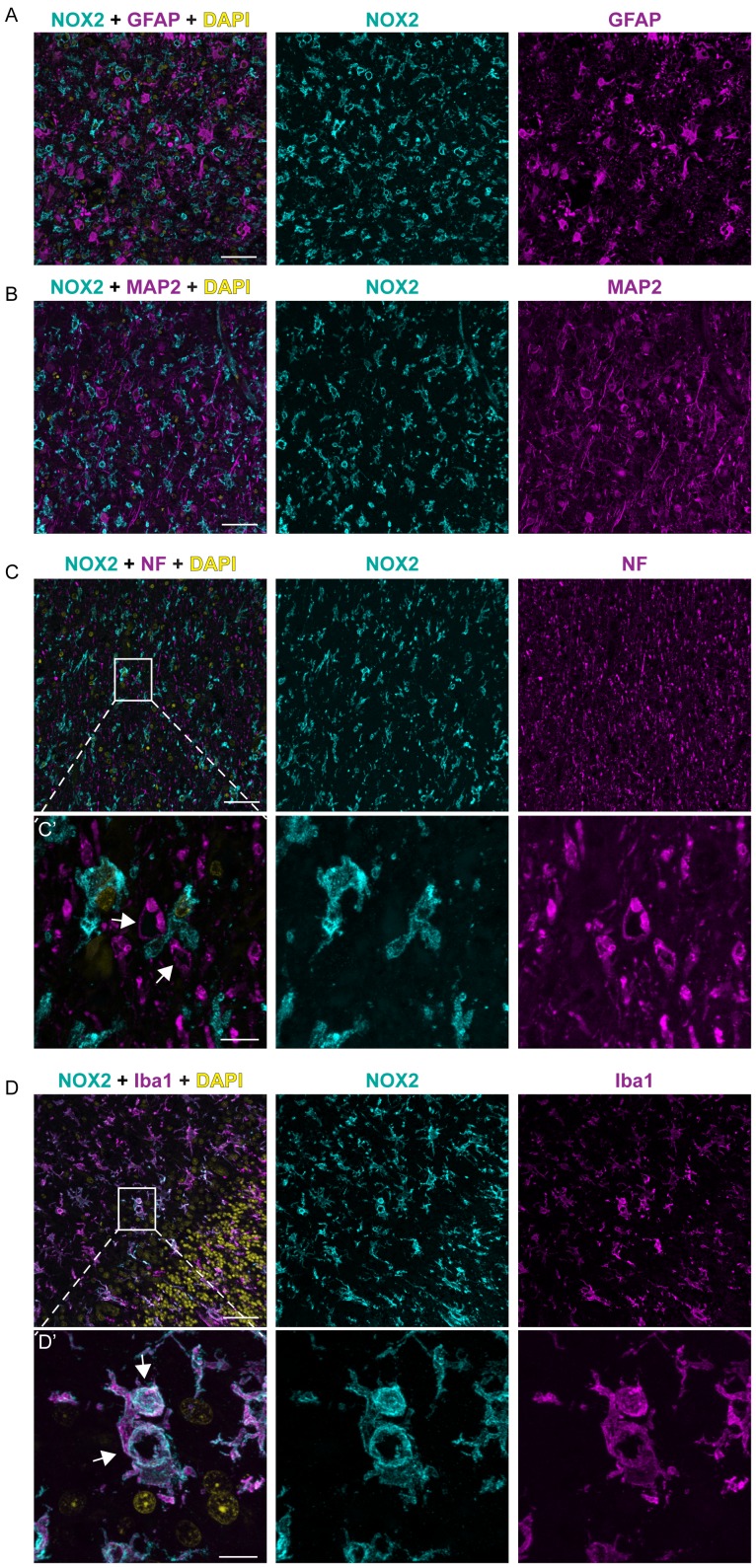
NOX2 expression is localized in microglia cells within CJD brains. Confocal images of immunofluorescence stainings on CJD patient brain sections: NOX2 is displayed in cyan; GFAP, MAP2, NF and IBA1 in magenta; DAPI in yellow. Panels **A**, **B**, **C**, **D**, from left to the right: overlay, NOX2 and GFAP, MAP2, NF, or IBA1, respectively; scale bar: 50 µm. Panels **C′** and **D′**: higher magnification of areas in C and D (indicated by box); from left to the right: overlay, NOX2 and NF or IBA1, respectively; arrows indicate spongiform vacuoles; scale bar: 10 µm.

In order to clarify the cellular localization of NOX2 in the brain of CJD patients, we performed multicolor immunofluorescent analyses using astrocytic (glial fibrillary acidic protein; GFAP), neuronal (microtubule-associated protein 2 (MAP2) and neurofilament heavy 200 kD subunit (NF)) as well as microglial markers (ionized calcium-binding adapter molecule 1; IBA1). Confocal imaging revealed no colocalization between NOX2 and either GFAP, MAP2 or NF (Fig. 2A–C). NF was also detected around a subset of vacuoles (Fig. 2C, lower set of panels), but the vacuoles strongly stained with NF antibodies were not superimposable with those stained for NOX2. Instead, cellular colocalization was detected between NOX2 and the microglial marker IBA1 (Fig. 2D). Strikingly, strong NOX2 immunoreactivity was present in microglial cells surrounding spongiform vacuoles (Fig. 2D, lower set). These data indicate that microglial NOX2 was significantly upregulated in brains of CJD patients, suggesting its involvement in the neuropathological changes developing in human prion diseases.

### Expression of NOX2 in prion disease experimental models

**Figure 3:**
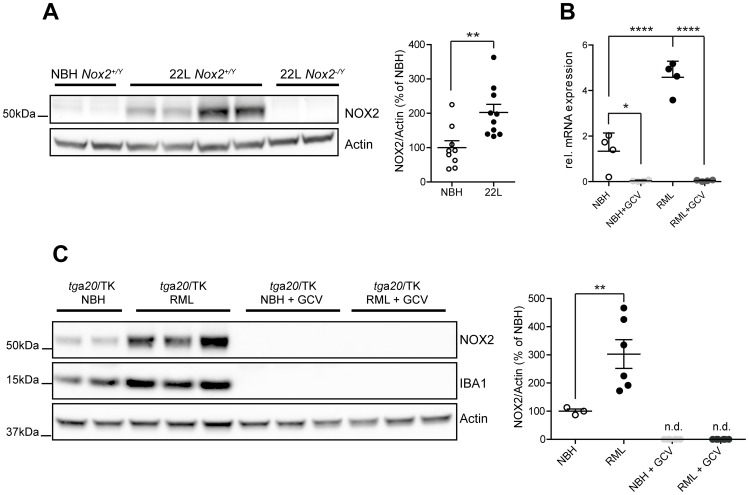
Analysis of NOX2 expression and cellular localization in mice. (**A**) Western blot of brain homogenates from *Nox2^+/Y^* and *Nox2^-/Y^* mice injected i.c. with prions (22 L) or non-infectious brain homogenate (NBH) using antibodies to NOX2 and Actin. Densitometric quantitation of NOX2 signal in *Nox2^+/Y^* samples was normalized over Actin signal. Scatter dot plot shows NOX2 relative signal intensity as percentage of NBH samples; each dot corresponds to a mouse (NBH, *n* = 9; 22 L, *n* = 10; **, P = 0.0044; Student's t test). (**B**) Quantitative RT-PCR analysis of *Nox2* expression in cerebellar slice cultures from *tg*a*20*/TK pups treated with NBH or prions (RML), with or without addition of ganciclovir (GCV). Significantly reduced *Nox2* expression levels are detected upon microglia depletion with ganciclovir; each dot corresponds to a pool of 6–9 cerebellar slices cultured in the same well (*n* = 4 pools; *, P<0.05; ****, P<0.0001; one-way ANOVA followed by Bonferroni's *post-hoc* test). (**C**) Western blot of cerebellar slice homogenates from *tg*a*20*/TK pups treated with NBH or prions (RML), with or without addition of GCV using NOX2, IBA1 or Actin antibodies. Densitometric quantitation of NOX2 signal was normalized over Actin signal. Scatter dot plot represents NOX2 relative signal intensity as percentage of NBH samples; each dot corresponds to a pool of 6-9 cerebellar slices cultured in the same well (NBH, *n* = 3 pools; RML, *n* = 6 pools; NBH+GCV, *n* = 6 pools; RML+GCV, *n* = 6 pools; **, P<0.001; one-way ANOVA followed by Bonferroni's *post-hoc* test).

We next analyzed by Western blotting the expression of NOX2 in mice inoculated with prions and sacrificed at the terminal stage of disease, and compared it with mice injected with non-infectious brain homogenate (NBH). NOX2 expression was significantly increased. The specificity of the signal was confirmed by absence of the relevant bands from brain homogenates of prion-inoculated, terminally sick *Nox2* deficient mice (Fig. 3A). Also in prion-infected organotypic cerebellar slices, we found that both NOX2 mRNA and protein levels were significantly increased over those of NBH-exposed slices (Fig. 3B–C). Hence NOX2 is significantly induced upon prion infection both *in vivo* and in cerebellar slices.

As a powerful tool to clarify the cellular localization of NOX2 also in mice, we took advantage of *CD11b-HSVTK* transgenic mice, which express the Herpes simplex thymidine kinase (*HSVTK*) under the transcriptional control of the *Itgam* promoter, active in macrophages and microglia. Administration of ganciclovir to these mice leads to selective microglia depletion [18]. Efficient microglia depletion, induced by ganciclovir treatment and confirmed by absence of IBA1 signal (Fig. 3C), led to complete suppression of *Nox2* transcript expression (Fig. 3B) and complete disappearance of all NOX2 protein (as assessed by Western blotting) from both NBH and prion-treated slices (Fig. 3C). These data indicate that NOX2 expression is linked to the presence of microglial cells.

### Effect of NOX2 on microglial proliferation, ROS production and spongiform changes after prion inoculation

Having assigned the expression of NOX2 to microglial cells, we investigated whether there would be a difference in microglial proliferation between wild-type and NOX2-deficient animals after prion inoculation. Since *Nox2* gene targeting was originally performed in 129-derived embryonic stem cells (CCE.1 line), with the resulting mice being backcrossed to the C57BL/6J genetic background [19], we assessed the extent and distribution of 129-derived genomic material in NOX2-deficient mice. Genome-wide single nucleotide polymorphism (SNP) analysis indicated that 99.86% of the analyzed SNPs were of C57BL/6 J type, with 129-derived SNPs clustering exclusively on the X chromosome in a region spanning <28.6 million base pairs comprising the *Nox2* locus. Extensive mapping studies on different combinations of mouse and prion strains failed to identify quantitative trait loci on the X chromosome significantly impacting prion incubation time [20]–[24]. Therefore, the 129-derived genomic region on NOX2-deficient mice is unlikely to represent a significant genetic confounder for our study [25].

We first verified that NOX2 deficiency did not affect PrP^C^ expression levels (S1 A-B Figure) and that partially proteinase-resistant prion protein accumulation occurred in both wild-type and *Nox2* knock-out animals (S1 C–D Figure).

**Figure 4:**
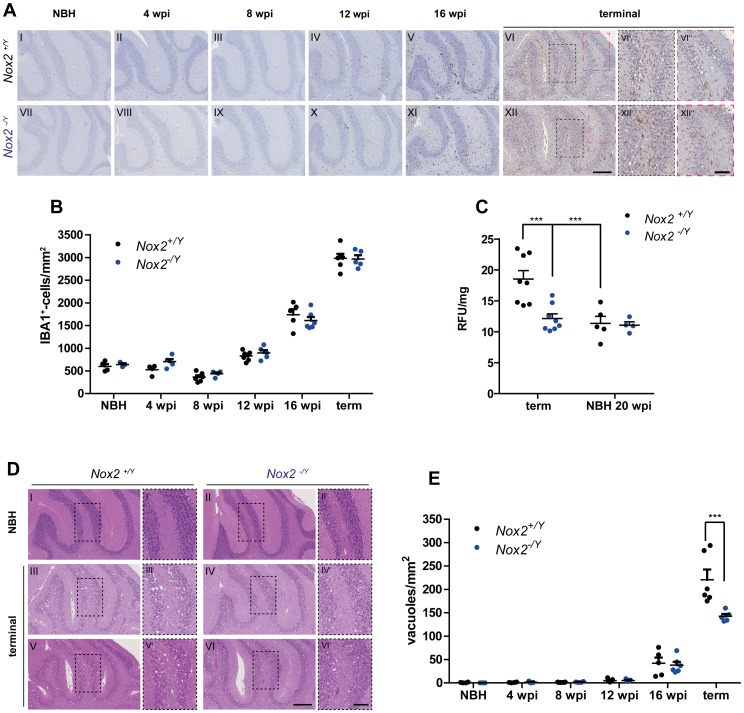
Effect of NOX2 expression on microglial proliferation, ROS production and spongiform changes after prion inoculation. (**A**) Microglial proliferation was analyzed by immunohistochemistry with the IBA1 antibody on brains of *Nox2^+/Y^* (A I-VI) and *Nox2^-/Y^* (A VII-XII) mice injected i.c. with NBH or prions and culled at 4, 8, 12, and 16 weeks post inoculation (wpi) or at the terminal stage of disease. Cerebellar areas are displayed in the pictures. Scale bar for A I-XII: 250 µm (displayed in panel XII). Dashed boxes in images VI and XII are shown at higher magnification in panels VI′-VI″ and XII′-XII″; scale bar: 100 µm (displayed in panel XII″). (**B**) Microglia cell number quantification in cerebellar cortex of *Nox2^+/Y^* and *Nox2^-/Y^* mice injected i.c. with NBH or 22 L prions and culled at different time points during disease incubation or at terminal stage. Each dot corresponds to one mouse (average of 3–5 sections per mouse). *Nox2^+/Y^*, *n* = 4–7; *Nox2^-/Y^*, *n* = 3–6; no significant difference between *Nox2^+/Y^* and *Nox2^-/Y^* mice; two-way ANOVA followed by Bonferroni's *post-hoc* test. (**C**) Detection of ROS production *in vivo* was performed by injecting i.p. the DHE probe and analyzing the fluorescence of its oxidation products in cerebellar homogenates. Dot plots show relative fluorescent units (RFU) per mg of proteins; each dot corresponds to one mouse (*Nox2^+/Y^*, *n* = 5–8; *Nox2^-/Y^*, *n* = 4–8): ***, P<0.001; two-way ANOVA followed by Bonferroni's *post-hoc* test. (**D**) Representative images of hematoxylin and eosin-stained sections from *Nox2^+/Y^* and *Nox2^-/Y^* mice injected i.c. with NBH or 22 L prions. Scale bar for D I-VI: 250 µm (displayed in panel VI). Dashed boxes in images I to VI are shown at higher magnification in panels I′-VI′; scale bar: 100 µm (displayed in panel VI′). (E) Number of spongiform vacuoles was quantified in cerebellar cortex of *Nox2^+/Y^* and *Nox2^-/Y^* mice injected i.c. with NBH or 22 L prions and culled at different time points during disease incubation or at terminal stage. Each dot corresponds to one mouse (average of 7–12 sections per mouse). *Nox2^+/Y^*, *n* = 4–7; *Nox2^-/Y^*, *n* = 3–6; ***P<0.0001; two-way ANOVA followed by Bonferroni's *post-hoc* test.

The extent of microglial reactions was analyzed by immunohistochemistry and Western blotting using the IBA1 antibody in male *Nox2^+/Y^* and *Nox2^-/Y^* littermates inoculated with 7 log LD_50_ units of the 22 L strain of prions. In agreement with previous reports [26], microglia immunostaining increased during disease incubation and became markedly prominent at the terminal stage (Fig. 4A). Quantification of IBA1 staining did not show a significant difference between *Nox2^+/Y^* and *Nox2^-/Y^* mice at any time point analyzed (Fig. 4B and S2A Figure). This observation was confirmed by Western blotting analysis (S3A Figure). Similarly, we could not see any effect on astrocytic response (S4A and B Figure).

Although NOX2 deficiency had no discernible influence onto the extent of microglia proliferation, it might still impact the production of ROS. We therefore investigated possible differences in oxidative stress levels by using *in vivo* injection of the dihydroethidium (DHE) probe. This molecule reacts with ROS and is converted into fluorescent adducts which can be measured in brain sample homogenates [27]. As expected from previous investigations, signs of oxidative stress production were identified in prion-infected, terminally scrapie-sick mice, but not in mice injected with normal brain homogenate. Excessive ROS production was only detected at the most advanced stages of disease, and was completely absent from NOX2-deficient mice (S3B Figure; Fig. 4C).

In addition, we found fewer spongiform changes in *Nox2*-deficient mice (Fig. 4D–E and S2B Figure). These results point to NOX2 as a major source of ROS in prion disease and a mediator of prion-induced neuronal toxicity.

### Effect of NOX2 expression on clinical manifestations of disease

**Figure 5:**
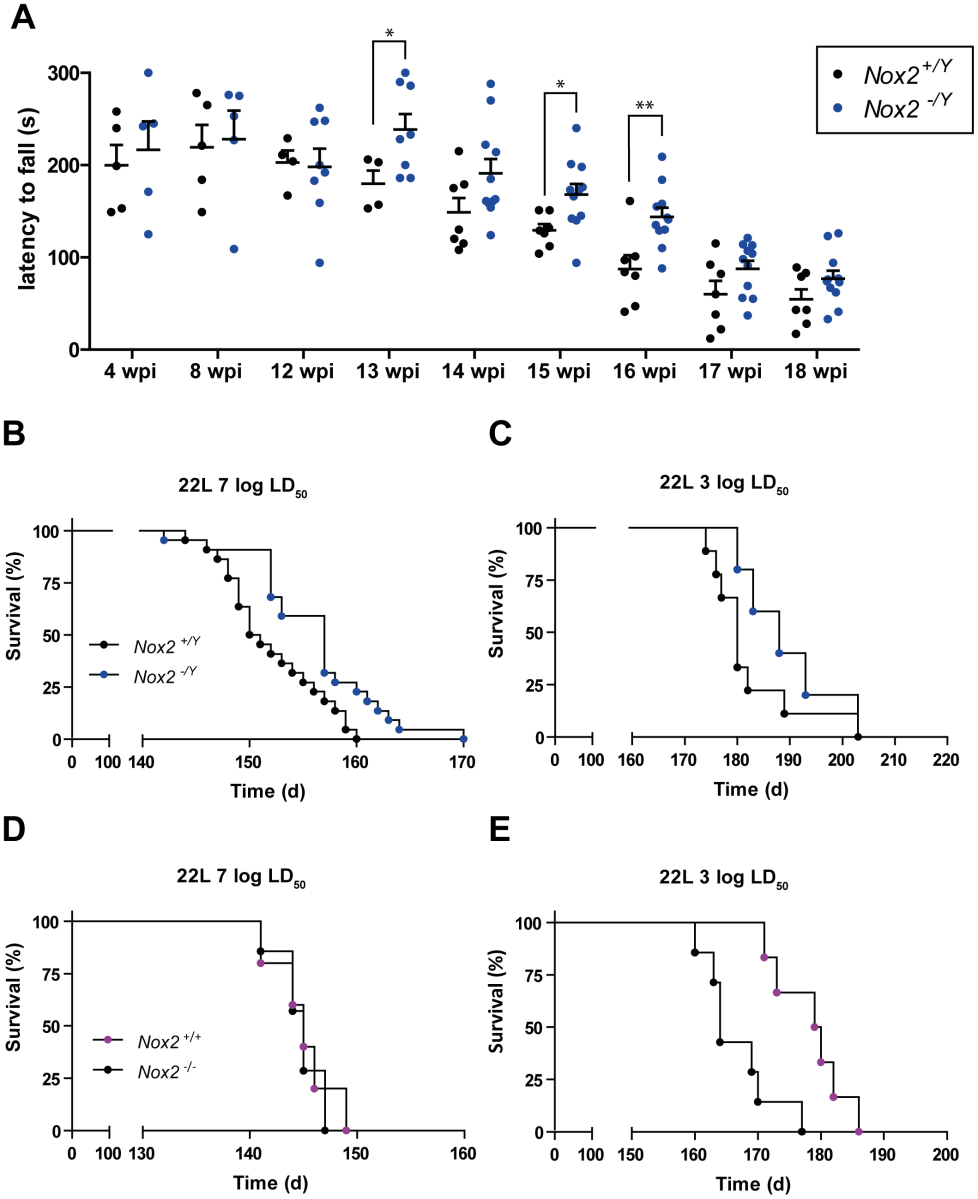
NOX2 deficiency attenuates the progression of prion disease. (**A**) Motor capacities of *Nox2^+/Y^* and *Nox2^-/Y^* mice were assessed with the rotarod test at specified time points after i.c. inoculation with 7 log LD_50_ units of 22 L prions. Scatter dot plot shows the time spent by each mouse on the rotating rod (latency to fall) expressed in seconds (s). Each dot corresponds to a mouse; a significant overall genotype effect (P = 0.025) was detected using a general linear mixed model. Student's t test per each time point also revealed a significant difference at 13 wpi (*, *p* = 0.0483); 15 wpi (*, *p* = 0.0246); and 16 wpi (**, *p* = 0.0048); *Nox2^+/Y^*, *n* = 4–7; *Nox2^-/Y^*, *n* = 5–11. (**B–C**) Survival curves of *Nox2^+/Y^* and *Nox2^-/Y^* males, inoculated i.c with 7 log LD_50_ (*Nox2^+/Y^*, *n* = 22, median incubation time 150.5 days post inoculation (dpi), *Nox2^-/Y^*, *n* = 22, 157 dpi; P = 0.0052; log-rank test) or 3 log LD_50_ (*Nox2^+/Y^*, *n* = 9, median incubation time 180 dpi, *Nox2^-/Y^*, *n* = 5, 188 dpi; P = 0.2176; log-rank test) units of 22 L prions. (**D–E**) Survival curves of females *Nox2^+/+^* and *Nox2^-/-^* inoculated i.c. with 7 log LD_50_ (*Nox2^+/+^*, *n* = 5, 145 dpi, *Nox2^-/-^*, *n* = 7, 145 dpi; P = 0.7289; log-rank test) or 3 log LD_50_ (*Nox2^+/+^*, *n* = 7, 164 dpi, *Nox2^-/-^*, *n* = 6, 179.5 dpi; P = 0.0030; log-rank test) units of 22 L prions.

In order to assess the neurological deficits induced by prion disease, we used the rotarod test, which primarily measures motor coordination and balance abilities [28]. Rotarod performance was similar in wild-type and NOX2-deficient mice at the early stage of the disease and up to 12 weeks following prion inoculation (Fig. 5A). Also, control mice injected with NBH did not display any difference during the entire test period, up to 20 weeks post-injection (S5 Figure), confirming previous reports that absence of NOX2 does not alter motor capacities [29].

In contrast, starting from 13 weeks post prion inoculation, we noticed a clear difference between the two groups. Wild-type animals displayed reduced motor capacities, being able to remain on the rotating rod for significantly shorter periods of time than NOX2-deficient mice (Fig. 5A). However, this improvement was transient, and was no longer detectable during the late stages of the disease (17–18 weeks post-inoculation). Nevertheless, NOX2 deficient mice enjoyed modest yet significantly increased survival (Fig. 5B–C). The median survival for *Nox2^+/Y^ vs. Nox2^-/Y^* mice was, respectively, 150.5 *vs.* 157 days post inoculation (dpi) after injection with 7 log LD_50_ units of prions and 180 *vs.* 188 dpi after inoculation with 3 log LD_50_ units (P = 0.0052 and 0.2176 respectively, log-rank test).

Similar to a previous report [30], we detected a difference in incubation time between males and females with both prion titers. Upon exposure to the 22 L strain of prions, wild-type females displayed accelerated progression of the disease as compared with wild-type males (7 log LD_50_ units =  median survival males 150.5 dpi, females 145 dpi; P<0.0001; 3 log LD_50_ units =  males 180 dpi, females 164 dpi; P = 0.0003, log-rank test). Possibly due to this accentuated effect on females, we only noticed a difference between wild-type and NOX2-deficient females after inoculation with lower doses of prions (Fig. 5D–E): the median survival for *Nox2^+/+^ vs. Nox2 ^-/-^* females was, respectively, 164 *vs.* 179.5 dpi after inoculation with 3 log LD_50_ units of prions, while was 145 dpi for both groups after inoculation with 7 log LD_50_ units (P = 0.0030 and P = 0.7289 respectively, log-rank test).

## Discussion

We have analyzed the role of NOX2 in a mouse model of prion disease. Our results indicate that NOX2, expressed in microglial cells, is a relevant source of oxidative stress in this context, and contributes to prion-induced functional alterations. Prion inoculation of mice faithfully reproduces the neuropathological and clinical manifestations of the corresponding human pathology, and indeed, a marked microglial NOX2 staining was present in brain sections of patients affected by CJD. In light of the recently recognized differences between human and murine microglia [31], the consistency of our findings in both species supports the validity of our results across multiple species including humans.

It has been reported that CJD patients experience higher levels of microglia proliferation and increased oxidative stress than patients suffering from Alzheimer's disease (AD) [10]. Analogously, we found stronger NOX2 staining in brain sections of CJD patients than in those of AD patients. Formation and accumulation of misfolded proteins is the main pathogenic event in several phenotypically diverse neurodegenerative diseases [32]. Such aggregates trigger a number of toxic and reparative responses, such as microglia activation, impaired neurotransmission and neuronal damage [33]. Signs of oxidative stress have been consistently detected among these processes, yet were mainly attributed to mitochondrial dysfunctions [34]. Instead, our data support a role for NOX2 enzyme as possible major source of ROS in protein misfolding diseases [15]. Therefore, beyond their relevance to the understanding of prion pathogenesis, our findings corroborate the idea that NOX2 inactivation is broadly implicated in neurodegenerative diseases. Moreover, immunohistochemistry for NOX2 proved to be a robust and reliable tool to visualize microglia, and might be the best among the available immunohistochemical markers for this cell type.

Intriguingly, we found a very striking pattern of NOX2 expression around neuronal vacuoles. Spongiform vacuolation is a highly characteristic sign of prion disease pathology, and vacuoles represent distended intracellular compartments mostly located within neuronal processes. While the mechanisms underlying the development of vacuoles in prion diseases have not been identified, the intracellular localization of vacuoles in prion diseases sets them apart from those observed in other neurological pathologies, such as AD or brain edema [35], [36]. Neurofilament proteins can abnormally accumulate around spongiform vacuoles [36], but it has also been reported that microglia can surround the rim of such vacuoles [37], [38]. The intimate topographic relationship between vacuoles and microglial processes suggest that the latter may respond to activating signals emanating directly from the vacuolated structures.

The cellular localization of NOX2 has been long debated. It has been suggested that NOX2 is expressed by neurons, yet most such evidence derives from *in vitro* studies or from immunostaining performed in absence of pertinent controls [15], [39]. Confocal imaging of both human brain tissues, as well as microglia depletion of murine organotypic slice cultures, indicates that within the brain, NOX2 expression is mainly restricted to microglial cells. Targeting NOX2 in microglial cells may selectively control deleterious functions exerted by microglia [40]. Moreover, NOX2 may be involved in the recently described microglia-regulated neuronal differentiation and circuitry remodeling during neurogenesis and synapse formation [41]-[43]. Differentiation of neural stem cells and synaptic plasticity can be indeed influenced by NOX2-dependent ROS production [15], which could therefore depend on NOX2 microglial rather than neuronal expression.

As previously reported for other pathologies [44], [45], NOX2 deficiency does not affect proliferation of microglia cells or accumulation of misfolded protein aggregates. Microglia-mediated phagocytosis is a crucial mechanism of defense against PrP^Sc^ deposition and prion-induced damage [46], [47]. Indeed, microglia removal by ganciclovir in *CD11b-HSVTK* slices led to increased levels of PK-resistant PrP^Sc^ [46]. Similarly, genetic ablation of the gene encoding for the milk fat globule–epidermal growth factor 8 (Mfge8), impaired the engulfment of apoptotic cells and was associated with augmented PrP^Sc^ accumulation. Since Mfg8 is secreted by astrocytes, while its receptors are expressed by microglia, it is possible that microglia-mediated phagocytosis serves to limit prion deposition. However, microglia overactivation can transform them in “saboteurs”, further contributing to the progression of the disease [48]. One of the mechanisms associated with this transformation can be the excessive production of NOX2-dependent ROS [49].

At the terminal stage of disease, NOX2 ablation completely prevented ROS production in prion-infected brains, which may represent a plausible explanation for the observed reduced spongiform changes and improved survival. In the early preclinical stages of the disease (4 to 12 weeks post-inoculation), we did not observe any difference between wild-type and NOX2-deficient animals in terms of ROS production and motor performance. However, starting from 13 weeks post inoculation, rotarod analysis revealed a significantly attenuated decline in locomotor abilities in NOX2-deficient mice, lasting up to 16 weeks post-inoculation. Interestingly, when ROS levels were analyzed at this time point, they were still indistinguishable from wild-type and NOX2-ablated mice. One possible explanation is that even very small amounts of ROS, below the detection threshold of the dihydroethidium assay, may play a role in neurological degradation following prion infections. Accordingly, increased levels of oxidative stress in the cerebellum were only found at the terminal stage of mouse scrapie [11].

Although NOX2 ablation does not ultimately prevent the development of prion disease, the results presented above show that NOX2 is a relevant constituent of the neurotoxic cascade in these diseases. Ablation or overexpression of superoxide dismutase, SOD1, activity can decrease or increase prion incubation time, respectively [7], [12]. Together with these previous findings, our data support the crucial impact of superoxide production in prion diseases. Moreover, they suggest that NOX2, expressed in microglial cells, is the major source of this superoxide production. The use of antioxidants as possible therapeutic approach for CNS diseases has been favored by promising results of rodent studies; however, disappointing and incongruous outcomes have been observed in clinical trials [50], [51]. Lack of specificity of antioxidant treatments could be one of the reasons that explain such a failure in clinical translation. Having identified NOX2 as a possible specific target may offer an opportunity to reduce the occurrence of oxidative stress insults in CJD patients. In particular, our data suggest that inhibition of NOX2 may attenuate, at least temporarily, the neurological dysfunctions associated with prion disease, thereby enhancing the quality of life – a legitimate and important goal even if the overall life expectancy may not be dramatically improved.

## Materials and Methods

### Ethics statement

All human tissue samples used in this study dated from before the year 2005, and were irreversibly anonymized. Approval by an institutional review board is not mandatory for irreversibly anonymized samples collected before the approval of the Swiss Medical-ethical guidelines and recommendations (Senate of the Swiss Academy of Medical Sciences, Basel, Switzerland, 23 May 2006). Animal care and experimental protocols were in accordance with the “Swiss Ethical Principles and Guidelines for Experiments on Animals”, and approved by the Veterinary office of the Canton of Zurich (permits 130/2008 and 41/2012). Prion inoculations were performed under isoflurane anesthesia, and every effort was made to minimize animal discomfort.

### Patients

Samples were obtained from the tissue bank of the Swiss National Reference Centre of Prion Diseases (Zurich, Switzerland). Samples from patients affected by dementia (CJD and AD) were used to quantify the extent of NOX2 expression. Samples from patients not affected by dementia were used as controls (S6 Figure). A subset of tissue specimens collected and analyzed according to biosafety guidelines outlined in our previous study [52] was used for this analysis.

### Mice

Congenic NOX2 knock-out mice [19] on a C57BL/6 J background were purchased from the Jackson Laboratory (B6.129S-*Cybb^tm1Din^*/J). For prion inoculations, littermates were obtained by breeding heterozygous *Nox2^+/-^* females with *Nox2^+/Y^* or *Nox2^-/Y^* males. For organotypic cerebellar slice cultures and microglia depletion experiments, *tg*a*20*/TK pups were obtained by breeding *tg*a*20* ^tg/tg^ *Prnp^o/o^* males on a B6129 mixed background to heterozygous congenic *CD11b-HSVTK* females on a C57BL/6 background [18]. For titration experiments, ICR (CD-1) and C57BL/6 mice were purchased from Harlan laboratories. Mice were bred in high hygienic grade facilities and housed in groups of 3–5, under a 12 h light/12 h dark cycle (from 7 am to 7 pm) at 21±1°C, with sterilized food (Kliba No. 3431, Provimi Kliba, Kaiseraugst, Switzerland) and water *ad libitum*.

### Whole-genome single nucleotide polymorphisms analysis

Genomic DNA was purified from tail biopsies using the Gentra Puregene Mouse Tail Kit (Qiagen) according to manufacturer's instructions. Whole-genome SNP analysis was performed using the Illumina Mouse MD Linkage Panel array (Taconic Laboratories) and results were compared with data from reference strains (129S6/SvEvTac, C57BL/6JBomTac, C57BL/6NTac).

### Prion inoculations

Inoculum of the 22 L strain of mouse-adapted scrapie prion was prepared from pooled 10% w/v brain homogenates of 22 L terminally sick CD-1 mice. Titration experiments were then performed by inoculating intracerebrally C57BL/6 recipients with serial dilutions of the 22 L inoculum. Infectivity titer (S2 Table) was calculated using the statistical method of Karber [53]. Wild-type and NOX2-deficient mice (8–10 weeks old) were injected intracerebrally with 30 µl of brain homogenate prepared in a solution of PBS/5% BSA, containing 7 log LD_50_ units or 3 log LD_50_ units of the 22L strain. Control mice received 30 µl of NBH derived from healthy CD-1 mice. Different sessions of inoculations were performed using aliquots of the same diluted inoculum depending on mouse availability. In each session littermates from both genotypes were used and allocation to experimental groups was performed before inoculation, in a randomized way. Prion-infected mice were sacrificed at 4, 8, 12 and 16 weeks post-inoculation (wpi) or at terminal stage, when they displayed typical clinical signs of the disease. The operator was blind to the genotypes.

### Rotarod

The rotarod test was used to assess motor coordination and balance at different time points after prion inoculations. The rotarod apparatus (Ugo Basile) consisted of five cylinders (3 cm diameter) separated by dividers (25 cm diameter) in five lanes, each 57 mm wide. During the habituation phase, mice were placed on the rotating drum (4 rpm lowest speed) for 3 sessions lasting 1–2 minutes each and separated by 10-minute intervals. Test phase started 30 minutes after the last habituation session, and consisted of 3 trials separated by 15-minute inter-trial intervals. Each test session lasted for a maximum of 5 minutes while the rod accelerated from 5 to 40 rpm. Latency to fall was determined when the mouse was no longer able to ride on the accelerating rod because slipping from the drum or clinging to the rod and rotating with it. Experiments were always performed at the same time of the day (between 10 am and 12 am), mice were tested in a randomized way and the operator was blind to the genotypes.

### Western blotting

Western blotting analyses were performed on brain tissue samples or COCS as previously described [46]. For specific detection of partially protease-resistant prion proteins PrP^Sc^, proteinase K (PK; Roche) digestion was performed (25 µg ml^−1^ PK for 20 µg protein lysate in a total volume of 20 µl). The following primary antibodies were used: NOX2 (1∶300, BD Bioscience), IBA1 (1: 5000; Wako); POM1 (200 ng ml^−1^; [54]): Actin (1∶15000, Millipore).

### Quantitative real-time PCR

Total RNA was extracted using the RNeasy Universal Plus Mini kit (QIAGEN) according to manufacturer's instructions. Genomic DNA was removed with the gDNA eliminator solution (provided in the same kit). cDNA was synthesized using 1 µg of RNA with the PrimeScript RT Reagent Kit with gDNA eraser (Takara Bio Inc.). Real-time PCR was performed in optical 384-well plates, in triplicates, using a Prism 7900 HT sequence detection system (Applied Biosystems) and the SYBR green master mix (Applied Biosystems). Raw Ct values were used to calculate relative expression levels of target genes, normalized according to geNorm analysis [55] to the housekeeping genes *Gapdh, Rps9* and *Eef1a1*. The following primers were used: *Nox2* forward 5′-CAGGAACCTCACTTTCCATAAGATG-3′; *Nox2* reverse 5′-AACGTTGAAGAGATGTGCAATTGT-3′; *Prnp* forward 5′-GCTGGCCCTCTTTGTGACTA-3′; *Prnp* reverse 5′-CTGGGCTTGTTCCACTGATT-3′; *Gapdh* forward 5′-TCCATGACAACTTTGGCATTG-3′; *Gapdh* reverse 5′-CAGTCTTCTGGGTGGCAGTGA-3′; *Rps9* forward 5′-GACCAGGAGCTAAAGTTGATTGGA-3′; *Rps9* reverse 5′-TCTTGGCCAGGGTAAACTTGA-3′; *Eef1a1* forward 5′-TCCACTTGGTCGCTTTGCT-3′; *Eef1a1* reverse 5′-CTTCTTGTCCACAGCTTTGATGA-3′.

### Microglia depletion

Cerebellar organotypic slices were prepared from 10–11-days-old *tg*a*20*/TK pups, treated with prions or NBH, cultured and processed for Western blotting or quantitative Real-Time PCR as previously described [46]. Microglia were depleted from slice cultures by adding ganciclovir from 0 to 21 days *in vitro* [18], [46]. Slices were harvested at 42 days post-inoculation for Western blotting or qPCR analyses (described above).

### PrP^C^-ELISA

PrP^C^ was quantified in brain tissue homogenate by sandwich ELISA using POM1 and POM2 antibodies as described previously [54].

### Histology and immunohistochemistry

Stainings were performed on sections from brain tissues fixed in formalin, treated with concentrated formic acid to inactivate prions, and embedded in paraffin. After deparaffinization through graded alcohols and heat-induced antigen retrieval in citrate buffer (0.01 M; pH 6), sections were incubated with the following antibodies: anti-human NOX2 (1∶250, Clone 48, Sanquin), GFAP (1∶1000, Millipore), IBA1 (1∶2500, WAKO), MAP2 (1∶500, Abcam), NF (1∶1000; Abcam). Single stainings were visualized using DAB (Sigma-Aldrich) and H_2_O_2_ (Sigma-Aldrich), after incubation with a biotinylated secondary antibody (Vector Laboratories) and the ABC complex solution (Vector laboratories). Hematoxylin counterstain was subsequently applied. Double stainings were performed with fluorescently-labeled secondary antibodies (1∶1000, Alexa Fluor 488 or 555; Invitrogen), followed by DAPI (Life technologies) nuclear staining. Partially protease-resistant prion protein deposits were visualized by staining brain sections with the SAF84 antibody (1∶200, SPI bio), on a NEXES immunohistochemistry robot (Ventana instruments) using an IVIEW DAB Detection Kit (Ventana), after preceding incubation with protease 1 (Ventana). Images of DAB stained sections were acquired using the NanoZoomer scanner (Hamamatsu Photonics) and NanoZoomer digital pathology software (NDPview; Hamamatsu Photonics). Quantifications of NOX2 staining in patient sections or IBA1, GFAP and vacuoles in mouse sections were performed on acquired images, where regions of interest were drawn on a Digital Image Hub (Leica Biosystems). The percentage of brown/white staining and the number of brown/white objects s over the total area were quantified using in-house–developed software. For the analyses, the computational algorithms were implemented using the C++ programming language and the OpenCV library (source code is available upon request). Operators were blind to the pathology and to the genotype of the analyzed sections. Images of fluorescent stainings were acquired with the confocal CLSM Leica SP5 (Leica Microsystems). Images were analyzed using the following image-processing softwares: Imaris (Bitplane), Adobe Photoshop and Adobe Illustrator.

### ROS detection

In order to measure the production of ROS *in vivo*, we used the DHE probe (Sigma-Aldrich) following a previously published protocol [27] with minor modifications. Mice were injected i.p. with 200 µl DHE (3 mM) solution. After 30 minutes of incubation, brain tissues were harvested, snap frozen in liquid nitrogen and stored at −80°C. Brain samples were homogenized in a buffer containing 50 mM KH_2_PO_4_, 1 mM EGTA and 150 mM sucrose. For quantification of DHE oxidation products, fluorescence was detected in 250 µl 2% (w/v) tissue sample homogenates using a fluorimeter (Ex/Em 485/585, cutoff: 570). Relative fluorescence units were normalized to protein concentration, determined by bicinchoninic acid assay (Pierce).

### Statistical analysis

Comparison between two groups was assessed with the two-tailed unpaired Student's t test, whereas multiple comparisons were assessed with the one-way ANOVA or two-way ANOVA followed by Bonferroni's *post-hoc* test. General linear mixed model was used to analyse rotarod data. Kaplan-Meier method was used to analyze incubation times and comparison between groups was made with the log-rank test. GraphPad and IBM SPSS Softwares were used for analysis and p-values <0.05 were considered statistically significant. For statistical analysis, logarithm transformation was performed of numerical data in fig. 1K, and square root transformation of numerical data in fig. 3B. The details of each analysis (statistical test, p-values, n) are indicated in the figure legends.

## Supporting Information

S1 Figure **PrP^C^ and PrP^Sc^ in** ***Nox2^+/Y^*** **and** ***Nox2^-/Y^*** **mice.** (**A**) Quantitative RT-PCR analysis of *Prnp* expression in non-infected *Nox2^+/Y^* and *Nox2^-/Y^* cerebellar tissue (*n* = 5). No significant differences were observed between *Nox2^+/Y^* and *Nox2^-/Y^* mice. (**B**) Levels of PrP^C^ were quantified by ELISA on non-infected *Nox2^+/Y^* and *Nox2^-/Y^* cerebellar homogenates (*n* = 5). No significant differences were observed between *Nox2^+/Y^* and *Nox2^-/Y^* mice. (**C**) Immunolabeled brain sections from *Nox2^+/Y^* (I and III) and *Nox2^-/Y^* (II and IV) mice injected with NBH or 22 L prions at terminal stage using anti-PrP antibody SAF84, after protease treatment. Scale bar: 250 µm (displayed in IV). (**D**) Western blot of PK-digested brain homogenates from *Nox2^+/Y^* and *Nox2^-/Y^* mice injected i.c. with NBH or 22 L prions using anti-PrP antibody POM1 (Ctr, non-PK-digested brain homogenate).
(TIF)

S2 Figure **Areas of IBA1 staining and spongiform vacuoles in** ***Nox2^+/Y^*** **and** ***Nox2^-/Y^*** **mice.** (**A**) Scatter dot plot shows average IBA1 expression, quantified as the percentage of the surface occupied by the IBA1 staining over the total measured area in selected regions of cerebellar cortex of *Nox2^+/Y^* and *Nox2^-/Y^* mice injected i.c. with NBH or 22 L prions and culled at different time points during disease incubation or at terminal stage. Each dot corresponds to one mouse (average of 3–5 sections per mouse). *Nox2^+/Y^*, *n* = 4–7; *Nox2^-/Y^*, *n* = 3–6; no significant difference between *Nox2^+/Y^* and *Nox2^-/Y^* mice; two-way ANOVA followed by Bonferroni's *post-hoc* test. (**B**) Area of spongiform vacuoles was quantified in cerebellar cortex of *Nox2^+/Y^* and *Nox2^-/Y^* mice injected i.c. with NBH or 22 L prions and culled at different time points during disease incubation or at terminal stage. Each dot corresponds to one mouse (average of 7–12 sections per mouse). *Nox2^+/Y^*, *n* = 4–7; *Nox2^-/Y^*, *n* = 3–6; *P<0.05; two-way ANOVA followed by Bonferroni's *post-hoc* test.
(TIF)

S3 Figure **Microglial proliferation and ROS production during prion incubation in** ***Nox2^+/Y^*** **and** ***Nox2^-/Y^*** **mice.** (**A**) Western blots of cerebellar homogenates from *Nox2^+/Y^* and *Nox2^-/Y^* mice culled at 4, 8, 12, 16 wpi or at terminal stage using IBA1 and Actin antibodies. Densitometric quantitation of IBA1 signal was normalized over Actin signal. Corresponding scatter dot plots show signal intensities as percentage of *Nox2^+/Y^* sample mean; each dot corresponds to one mouse (*Nox2^+/Y^*, *n* = 4–5; *Nox2^-/Y^*, *n* = 5; no significant difference; Student's t test). (**B**) Detection of ROS production *in vivo* was performed by injecting i.p. the DHE probe and analyzing the fluorescence of its oxidation products in cerebellar homogenates. Scatter dot plot shows relative fluorescent units (RFU) per mg of proteins; each dot corresponds to one mouse (*Nox2^+/Y^*, *n* = 4–9; *Nox2^-/Y^*, *n* = 3–9); no significant differences were detected.
(TIF)

S4 Figure **Astrocyte reaction in** ***Nox2^+/Y^*** **and** ***Nox2^-/Y^*** **mice.** (**A**) Astrocyte reaction was analyzed by immunohistochemistry with the GFAP antibody in *Nox2^+/Y^* (A I-VI) and *Nox2^-/Y^* (A VII-XII) mice injected i.c. with NBH or prions and culled at 4, 8, 12, 16 weeks post inoculation (wpi) or at the terminal stage of disease. Cerebellar areas are displayed in the pictures. Scale bar: 250 µm (displayed in panel XII). (**B**) GFAP staining was quantified in cerebellar cortex of *Nox2^+/Y^* and *Nox2^-/Y^* mice injected i.c. with NBH or 22 L prions. Each dot corresponds to one mouse (average of 3–5 sections per mouse). *Nox2^+/Y^*, *n* = 3–6; *Nox2^-/Y^*, *n* = 3–5; no significant difference between *Nox2^+/Y^* and *Nox2^-/Y^* mice; two-way ANOVA followed by Bonferroni's *post-hoc* test.
(TIF)

S5 Figure **Rotarod performance of** ***Nox2^+/Y^*** **and** ***Nox2^-/Y^*** **mice injected with NBH.** Motor capacities of *Nox2^+/Y^* and *Nox2^-/Y^* mice were assessed with the rotarod test at 4, 8, 12, 16 and 20 weeks after injection (wpi) with NBH. Scatter dot plot shows the time spent by each mouse on the rotating rod (latency to fall) expressed in seconds (s). Each dot corresponds to a mouse; no significant differences were detected (*Nox2^+/Y^*, *n* = 5; *Nox2^-/Y^*, *n* = 4; Student's t test).
(TIF)

S6 Figure **NOX2 staining in non-demented cases.** Representative pictures of NOX2 staining in cortical tissue of patients with (**A**) and without (**B**) ischemic lesions. Scale bar: 100 µm.
(TIF)

S1 Table **Basic characteristics of CJD and control patients.**
(PDF)

S2 Table **22 L titration in C57BL/6 mice.**
(PDF)
