# Supplementary figures and images for: The Role of the NADPH Oxidase NOX2 in Prion Pathogenesis
Source: PLoS Pathog. 2014 Dec 11;10(12):e1004531. doi: 10.1371/journal.ppat.1004531 (PMC4263757; doi:10.1371/journal.ppat.1004531)

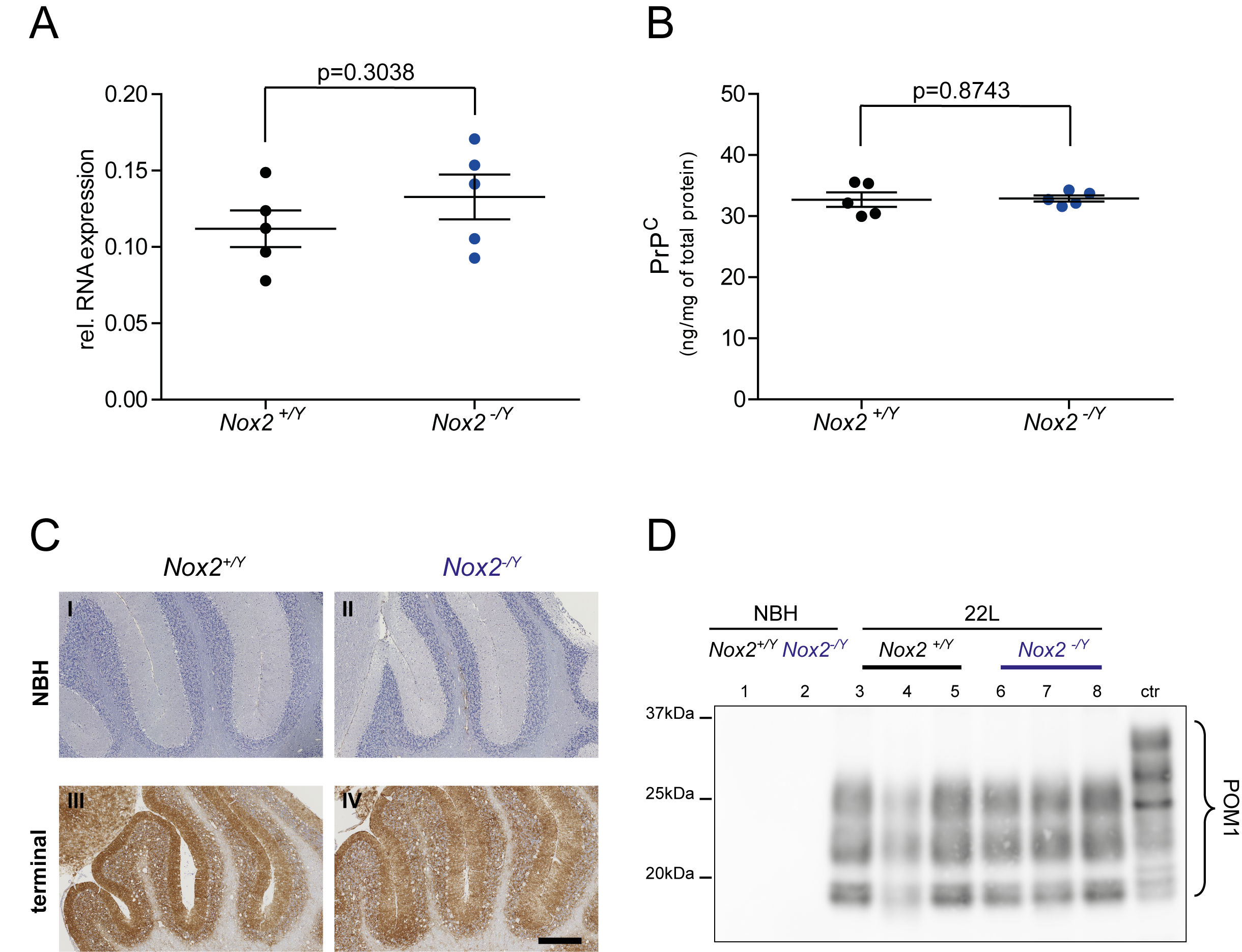

Supplement: S1 Figure — PrPC and PrPSc in Nox2+/Y and Nox2-/Y mice. (A) Quantitative RT-PCR analysis of Prnp expression in non-infected Nox2+/Y and Nox2-/Y cerebellar tissue (n = 5). No significant differences were observed between Nox2+/Y and Nox2-/Y mice. (B) Levels of PrPC were quantified by ELISA on non-infected Nox2+/Y and Nox2-/Y cerebellar homogenates (n = 5). No significant differences were observed between Nox2+/Y and Nox2-/Y mice. (C) Immunolabeled brain sections from Nox2+/Y (I and III) and Nox2-/Y (II and IV) mice injected with NBH or 22 L prions at terminal stage using anti-PrP antibody SAF84, after protease treatment. Scale bar: 250 µm (displayed in IV). (D) Western blot of PK-digested brain homogenates from Nox2+/Y and Nox2-/Y mice injected i.c. with NBH or 22 L prions using anti-PrP antibody POM1 (Ctr, non-PK-digested brain homogenate). (TIF) [file ppat.1004531.s001.tif]

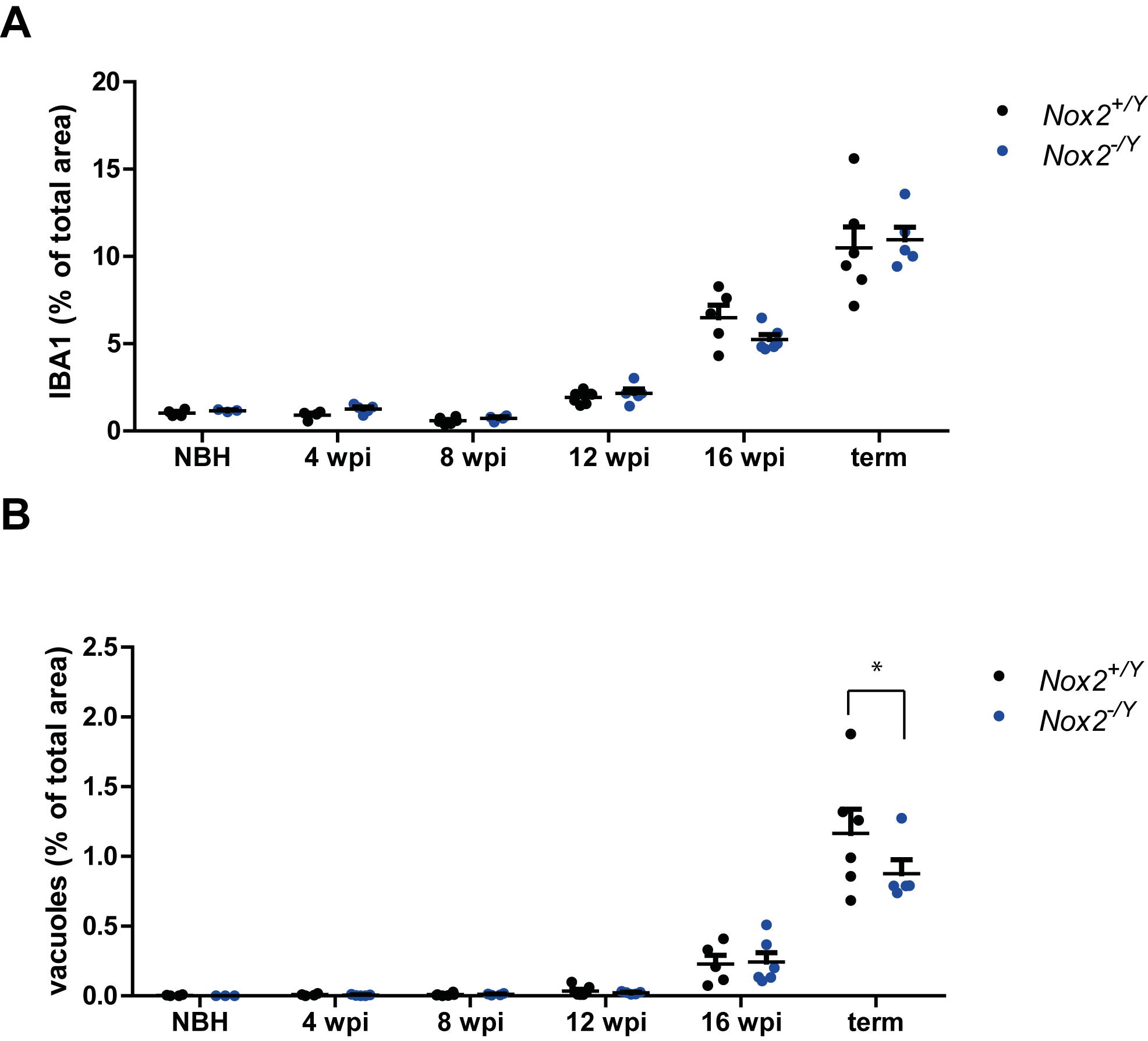

Supplement: S2 Figure — Areas of IBA1 staining and spongiform vacuoles in Nox2+/Y and Nox2-/Y mice. (A) Scatter dot plot shows average IBA1 expression, quantified as the percentage of the surface occupied by the IBA1 staining over the total measured area in selected regions of cerebellar cortex of Nox2+/Y and Nox2-/Y mice injected i.c. with NBH or 22 L prions and culled at different time points during disease incubation or at terminal stage. Each dot corresponds to one mouse (average of 3–5 sections per mouse). Nox2+/Y, n = 4–7; Nox2-/Y, n = 3–6; no significant difference between Nox2+/Y and Nox2-/Y mice; two-way ANOVA followed by Bonferroni's post-hoc test. (B) Area of spongiform vacuoles was quantified in cerebellar cortex of Nox2+/Y and Nox2-/Y mice injected i.c. with NBH or 22 L prions and culled at different time points during disease incubation or at terminal stage. Each dot corresponds to one mouse (average of 7–12 sections per mouse). Nox2+/Y, n = 4–7; Nox2-/Y, n = 3–6; *P<0.05; two-way ANOVA followed by Bonferroni's post-hoc test. (TIF) [file ppat.1004531.s002.tif]

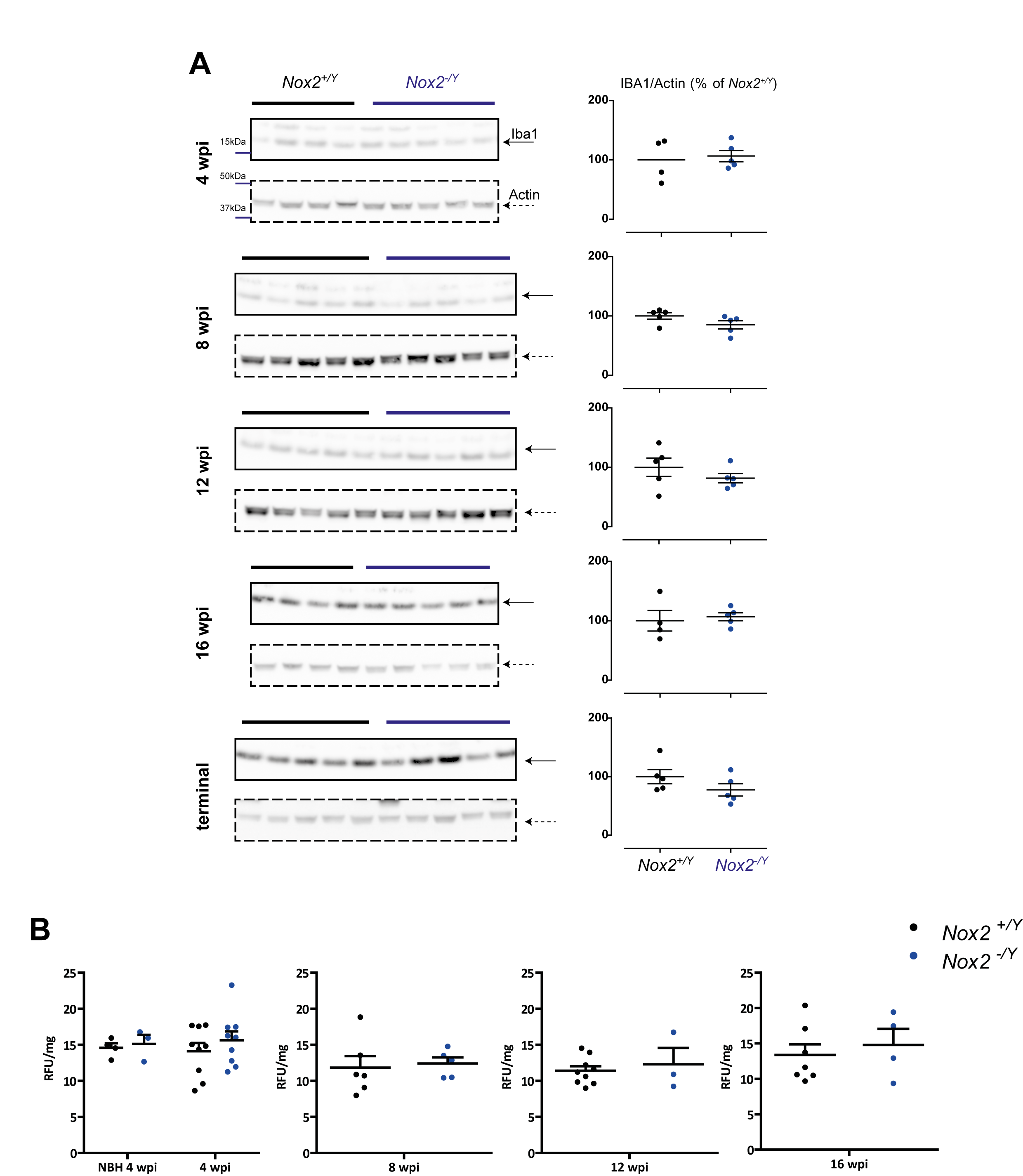

Supplement: S3 Figure — Microglial proliferation and ROS production during prion incubation in Nox2+/Y and Nox2-/Y mice. (A) Western blots of cerebellar homogenates from Nox2+/Y and Nox2-/Y mice culled at 4, 8, 12, 16 wpi or at terminal stage using IBA1 and Actin antibodies. Densitometric quantitation of IBA1 signal was normalized over Actin signal. Corresponding scatter dot plots show signal intensities as percentage of Nox2+/Y sample mean; each dot corresponds to one mouse (Nox2+/Y, n = 4–5; Nox2-/Y, n = 5; no significant difference; Student's t test). (B) Detection of ROS production in vivo was performed by injecting i.p. the DHE probe and analyzing the fluorescence of its oxidation products in cerebellar homogenates. Scatter dot plot shows relative fluorescent units (RFU) per mg of proteins; each dot corresponds to one mouse (Nox2+/Y, n = 4–9; Nox2-/Y, n = 3–9); no significant differences were detected. (TIF) [file ppat.1004531.s003.tif]

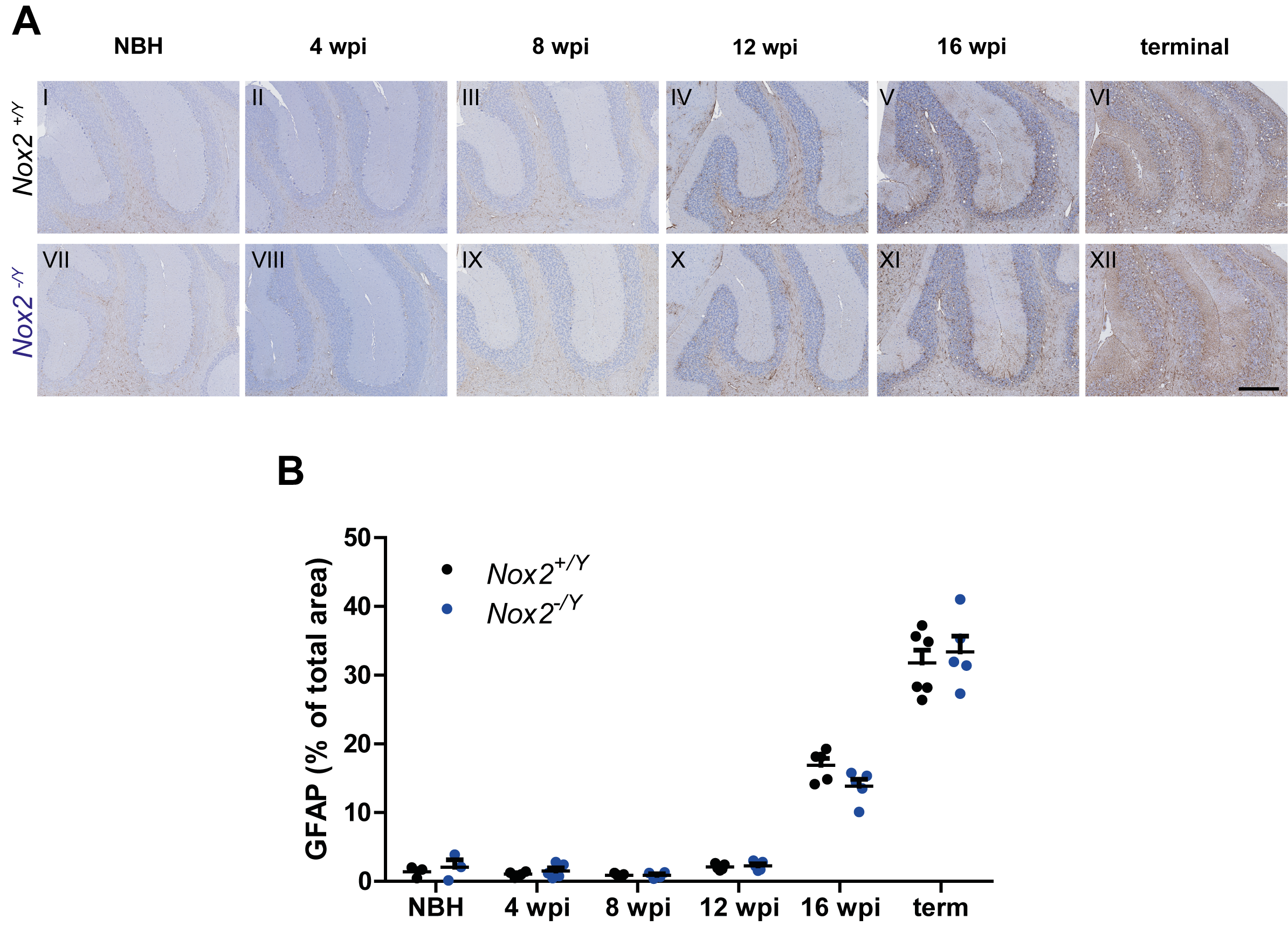

Supplement: S4 Figure — Astrocyte reaction in Nox2+/Y and Nox2-/Y mice. (A) Astrocyte reaction was analyzed by immunohistochemistry with the GFAP antibody in Nox2+/Y (A I-VI) and Nox2-/Y (A VII-XII) mice injected i.c. with NBH or prions and culled at 4, 8, 12, 16 weeks post inoculation (wpi) or at the terminal stage of disease. Cerebellar areas are displayed in the pictures. Scale bar: 250 µm (displayed in panel XII). (B) GFAP staining was quantified in cerebellar cortex of Nox2+/Y and Nox2-/Y mice injected i.c. with NBH or 22 L prions. Each dot corresponds to one mouse (average of 3–5 sections per mouse). Nox2+/Y, n = 3–6; Nox2-/Y, n = 3–5; no significant difference between Nox2+/Y and Nox2-/Y mice; two-way ANOVA followed by Bonferroni's post-hoc test. (TIF) [file ppat.1004531.s004.tif]

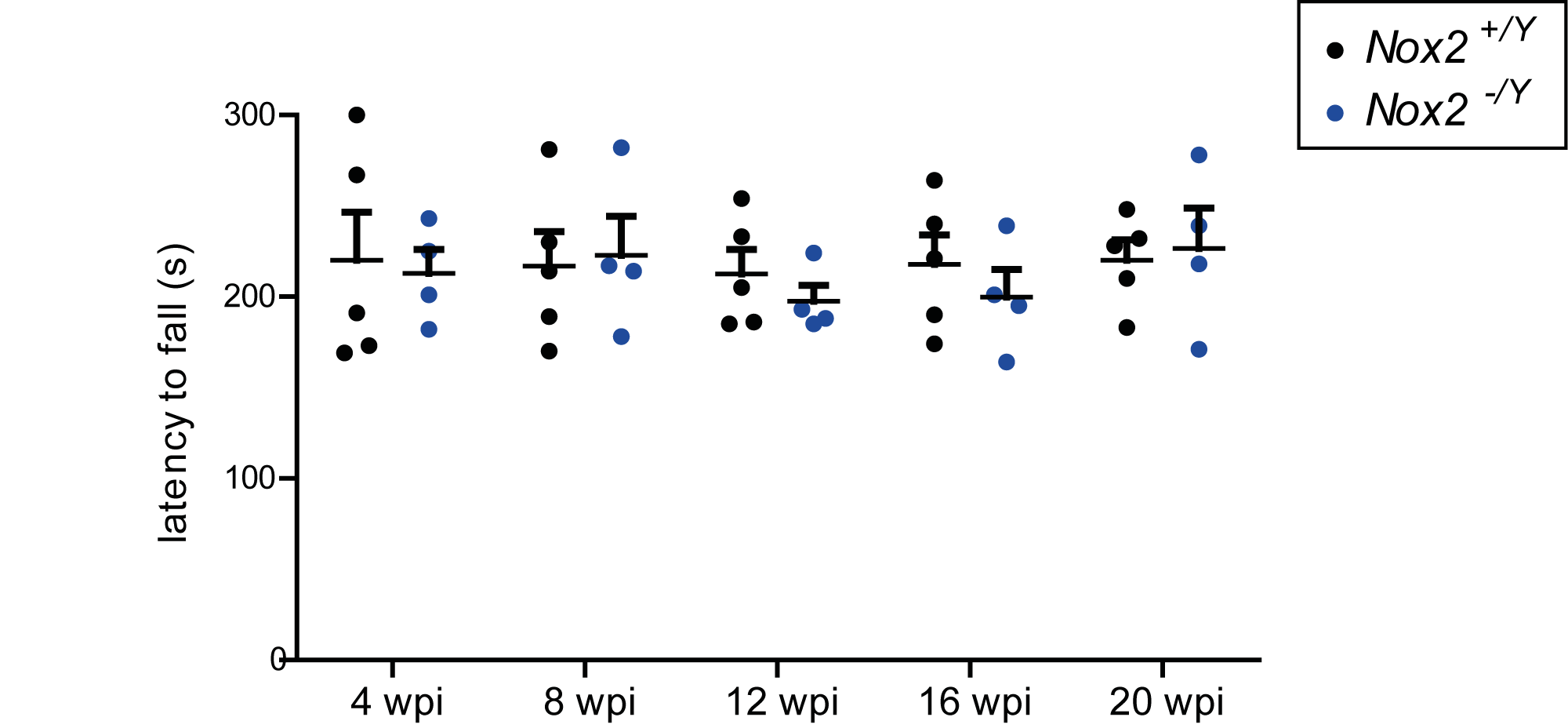

Supplement: S5 Figure — Rotarod performance of Nox2+/Y and Nox2-/Y mice injected with NBH. Motor capacities of Nox2+/Y and Nox2-/Y mice were assessed with the rotarod test at 4, 8, 12, 16 and 20 weeks after injection (wpi) with NBH. Scatter dot plot shows the time spent by each mouse on the rotating rod (latency to fall) expressed in seconds (s). Each dot corresponds to a mouse; no significant differences were detected (Nox2+/Y, n = 5; Nox2-/Y, n = 4; Student's t test). (TIF) [file ppat.1004531.s005.tif]

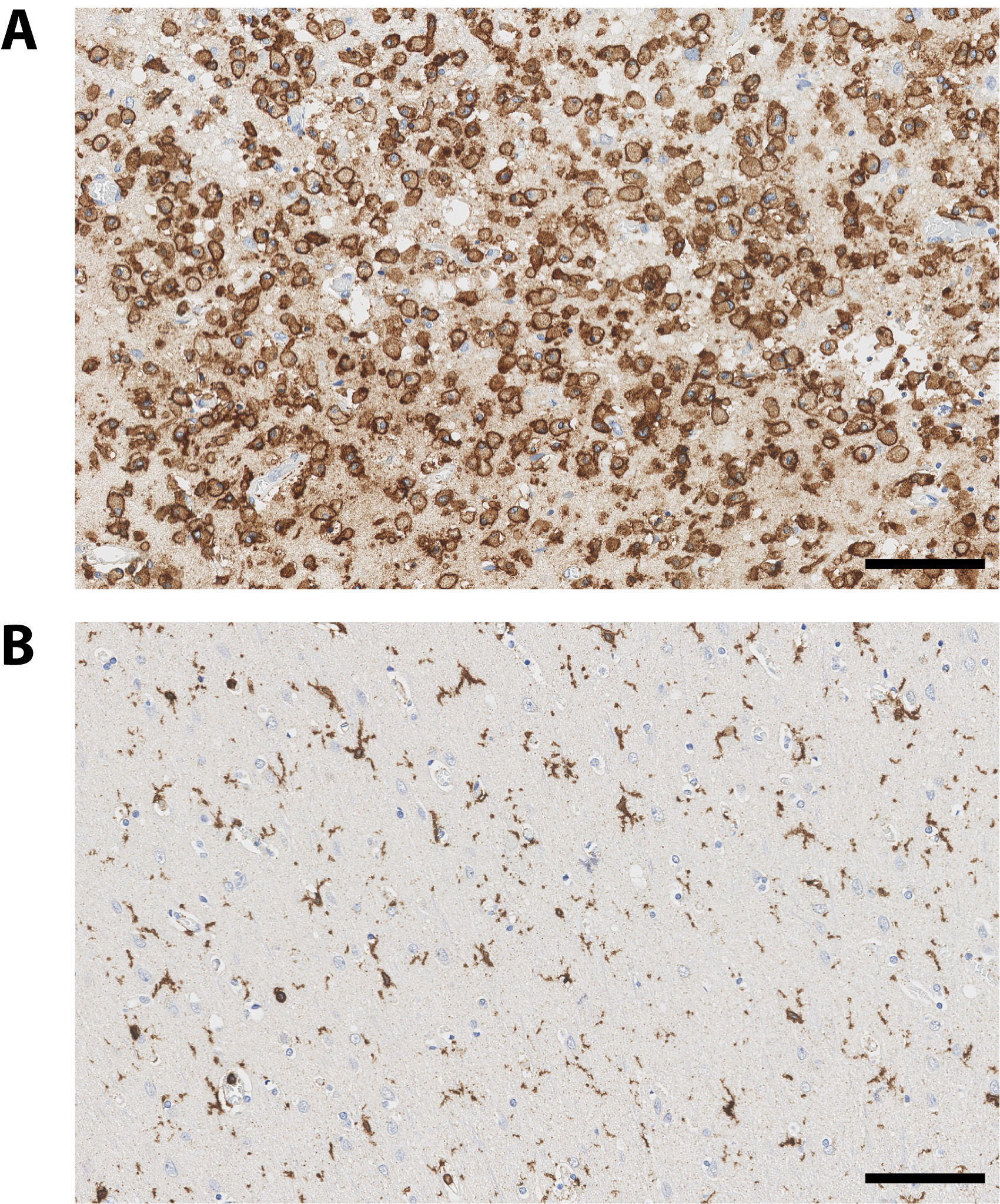

Supplement: S6 Figure — NOX2 staining in non-demented cases. Representative pictures of NOX2 staining in cortical tissue of patients with (A) and without (B) ischemic lesions. Scale bar: 100 µm. (TIF) [file ppat.1004531.s006.tif]
