# Supplementary material for: The Role of the NADPH Oxidase NOX2 in Prion Pathogenesis
Source: PLoS Pathog. 2014 Dec 11;10(12):e1004531. doi: 10.1371/journal.ppat.1004531 (PMC4263757; doi:10.1371/journal.ppat.1004531)
Supplement: S1 Table — Basic characteristics of CJD and control patients. (PDF) [file ppat.1004531.s007.pdf]

Table S1: Basic characteristics of CJD and control patients**.**

Patient n°	Gender	Age at death (years)	Duration of disease (months)	PrP^{Sc} type
1	M	68	n.a.	2
2	F	68	6	2
3	M	73	n.a.	2
4	M	48	8	2
6	M	68	5	2
5	F	81	10	1
7	M	41	2	1
8	M	84	9	1
9	M	63	3	1
10	M	75	7	1
C1	M	76	12	neg.
C2	M	84	n.a.	neg.
C3	F	75	14	neg.

n.a.= not available
