# Supplementary material for: The Role of the NADPH Oxidase NOX2 in Prion Pathogenesis
Source: PLoS Pathog. 2014 Dec 11;10(12):e1004531. doi: 10.1371/journal.ppat.1004531 (PMC4263757; doi:10.1371/journal.ppat.1004531)
Supplement: S2 Table — 22 L titration in C57BL/6 mice. (PDF) [file ppat.1004531.s008.pdf]

Table S2. 22L titration in C57BL/6 mice

Dilution	Sick/Total	Incubation time of recipient (days)	% mortality
10^{-5}	4/4	168,170,188,199	100
$10^{-5.5}$	4/4	170,172,181,188	100
10^{-6}	3/4	172,233,236,>349	75
$10^{-6.5}$	3/4	220,227,262,>349	75
10^{-7}	1/4	199,>337,>337,>337	25
$10^{-7.5}$	0/4	>337	0
10^{-8}	0/4	>337	0
$10^{-8.5}$	1/4	234,>300	25
10^{-9}	0/4	>300	0
		**8 log LD_{50} units/ml in 10% homogenate**	
